# Translation, adaptation and validation the contents of the Diabetes Medical Management Plan for the Brazilian context

**DOI:** 10.1590/1518-8345.1138.2740

**Published:** 2016-08-08

**Authors:** Heloísa de Carvalho Torres, Fernanda Figueredo Chaves, Daniel Dutra Romualdo da Silva, Adriana Aparecida Bosco, Beatriz Diniz Gabriel, Ilka Afonso Reis, Júlia Santos Nunes Rodrigues, Adriana Silvina Pagano

**Affiliations:** 1 PhD, Associate Professor, Faculdade de Enfermagem, Universidade Federal de Minas Gerais, Belo Horizonte, MG, Brazil.; 2 Master's Student, Escola de Enfermagem, Universidade Federal de Minas Gerais, Belo Horizonte, MG, Brazil.; 3 MSc, Regional Minas Gerais, Sociedade Brasileira de Diabetes, Belo Horizonte, MG, Brazil.; 4 PhD, Regional Minas Gerais, Sociedade Brasileira de Diabetes, Belo Horizonte, MG, Brazil.; 5 MSc, Regional Minas Gerais, Sociedade Brasileira de Diabetes, Belo Horizonte, MG, Brazil.; 6 PhD, Associate Professor, Minas Gerais, Sociedade Brasileira de Diabetes, Belo Horizonte, MG, Brazil.; 7 Master´s Student, Faculdade de Letras, Universidade Federal de Minas Gerais, Belo Horizonte, MG, Brazil.; 8 PhD, Full Professor, Faculdade de Letras, Universidade Federal de Minas Gerais, Belo Horizonte, MG, Brazil.

**Keywords:** Diabetes Mellitus, School Health, Questionnaires, Validation Studies, Committee Membership

## Abstract

**Objective:**

to translate, adapt and validate the contents of the Diabetes Medical Management Plan for the Brazilian context. This protocol was developed by the American Diabetes Association and guides the procedure of educators for the care of children and adolescents with diabetes in schools.

**Method:**

this methodological study was conducted in four stages: initial translation, synthesis of initial translation, back translation and content validation by an expert committee, composed of 94 specialists (29 applied linguists and 65 health professionals), for evaluation of the translated version through an online questionnaire. The concordance level of the judges was calculated based on the Content Validity Index. Data were exported into the R program for statistical analysis:

**Results:**

the evaluation of the instrument showed good concordance between the judges of the Health and Applied Linguistics areas, with a mean content validity index of 0.9 and 0.89, respectively, and slight variability of the index between groups (difference of less than 0.01). The items in the translated version, evaluated as unsatisfactory by the judges, were reformulated based on the considerations of the professionals of each group.

**Conclusion:**

a Brazilian version of Diabetes Medical Management Plan was constructed, called the Plano de Manejo do Diabetes na Escola.

## Introduction

Type 1 diabetes mellitus (DM1) is the second most common chronic condition in childhood and adolescence, presenting a world-wide increase in incidence of 3% per year, mostly in children under fifteen years of age^1^ ^-^ ^3^. Given this panorama, the adoption of strategies that facilitate diabetes management in schools has been suggested, in order to improve the care of these children and adolescents, with minimal disruption of the routine treatment, considering the fact that they spend much of the time within this environment^4^ ^-^ ^5^.

Studies claim that the planning of care in diabetes in schools helps in the control of blood sugar levels, improves confidence and minimizes the concern of both the family and the professionals of the educational institution^6^ ^-^ ^9^.

In this context, the American Diabetes Association (ADA) developed the Diabetes Medical Management Plan*, a protocol of procedures with clear and concise context that facilitate the process of communication between the health professionals, parents and educators and guide the care of children and adolescents with diabetes in the school^10^ ^-^ ^11^. The instrument contains specific and individual guidelines for the treatment of the diabetes condition to be followed in schools, being divided into the following sections: glucose monitoring, hypoglycemia treatment, hyperglycemia treatment, insulin therapy, insulin pump therapy, diet plan, and physical activities and sports^10^ ^-^ ^11^. In Brazil, there is no record of a similar official instrument that promotes safety, health and the inclusion of children and adolescents with diabetes in schools.

Given the need to provide a tool that can guide the education for the care of DM1 in Brazilian schools, the Brazilian Diabetes Society- Regional Minas Gerais (SBD-MG), in partnership with the School of Nursing, the Experimental Laboratory of Translation of the Faculty of Languages and the Laboratory of Biostatistics of the Federal University of Minas Gerais (UFMG), conducted this study. This was part of the Empoder@ project - methodological innovation in educational practices directed toward autonomy in healthcare, which aims to achieve a more enhanced control of this chronic condition and improve the quality of life of children and adolescents.

Several criticisms have been made regarding the conventional methodology of translation and adaptation of questionnaires in healthcare, as the translation by professional translators has been insufficient for the production of culturally appropriate questionnaires: the translation is made in an uninformed manner regarding the context in which the questionnaires will be used and no dialogue with the professionals directly involved in the care of the individuals^12^.

Accordingly, in order to provide a guidance protocol for diabetes management in schools, for use in the Brazilian context, the aim of this study was to translate and adapt the Diabetes Medical Management Plan and to validate the contents of its Brazilian version.

## Method

This methodological study was initiated after the approval of the American Diabetes Association to use, translate and adapt the original version of the Diabetes Medical Management Plan. The traditional methodological steps - initial translation, translation synthesis and back translation - were performed by the Experimental Laboratory of Translation of the Federal University of Minas Gerais Faculty of Languages, in partnership with the Brazilian Diabetes Society - Regional Minas Gerais, which generated the translated version of the instrument.

In the next step, a sample of professionals was selected to serve on the Committee of Judges to evaluate the translated version, consisting of professionals trained in Applied Linguistics and with translation experience and professionals of the Health area with experience in the healthcare and education of children and adolescents with diabetes. This was a convenience sample, and the Committee of Judges was formed from the invitation to 128 professionals of Health and 54 of Applied Linguistics, in a proportion of 70% and 30%, respectively, which is in accordance with the greater weight given to the evaluation by the health professionals, as the questionnaire is from their area of ​​expertise.

A letter of invitation was sent to the professionals, by e-mail, explaining the aims and methodology of the study, the justification for the process of translation, adaptation and validation of the content of the instrument, and the request for their participation in the study as evaluator judges through access to the platform web e-Surv**. If they did not respond to the questionnaire within seven days, a new e-mail and phone text message (if available) were sent to reinforce the invitation.

In the evaluation requested, the judges were asked to assign one of the four following adjustment options to each section of the translated instrument, compared to the original version: 1. one star - need for complete retranslation, 2. two stars - need for partial retranslation with many changes, 3. three stars - need for partial retranslation with optional changes to improve the text style, and 4. four stars - no need for retranslation. The judges were also requested to comment on the translated items, such as suggestions to improve the instrument.

The Content Validity Index (CVI), defined by the sum of the relative frequencies of the three- and four-star responses was calculated to verify the level of concordance of the judges in relation to the adequacy of the items evaluated. A CVI greater than or equal to 0.78 was considered indicative of adequacy to the original text, for both the evaluation of each item and for the general evaluation of the instrument^13^. With higher CVIs indicating fewer changes needed to improve the text style. The comments were considered important, according to the area of practice of the professional​​, whether Health or Applied Linguistics, considering the knowledge of each. 

All data were coded and stored anonymously in a spreadsheet, which was exported for analysis in the R statistical program. The project was approved by the Research Ethics Committee for Studies Involving Human Subjects of the Federal University of Minas Gerais, under authorization No. 1.020.023. The agreement of the professionals to freely participate in the study was recorded in the initial menu of the electronic questionnaire on the *e-Surv* platform.

## Results

Of the 182 invitations sent, 94 questionnaires were received via *e-Surv*, with a response rate of 51% and 54% in the areas of Health and Applied Linguistics, respectively. Of the 94 answered questionnaires, 65 were completed by professionals of Health (70%) and 29 by those of Applied Linguistics (30%). 

The demographic data of the judges and data related to their knowledge of the English language are presented in Table 1. Education at a *lato sensu* post-graduation level was registered for 40.4% of the judges, and 59.6% of the judges said they had completed a postgraduate s*tricto sensu* course. Of the professionals from both areas, 43.6% had MSc. or PhD., reinforcing a profile of academic education appropriate for them to contribute to this study. The majority of the judges declared that they understood the English language well, reaching the percentages of 72.4% in Health and 82.7% in Applied Linguistics. The same trend was observed for the ability to read the text in English, with a good level being declared by the majority of the judges, both of Health and Applied Linguistics (78.4 and 93.1%, respectively).

**Table 1:** Characterization of the professional participants of the Committee of Judges from the areas of Health and Applied Linguistics. Belo Horizonte, MG, Brazil, 2015

Professionals			N (%)*	
			Health	Applied Linguistics
Physicians			42 (64.6)	--
Nurses			10 (15.4)	--
Nutritionists			6 (9.3)	--
Physical educators			3 (4.6)	--
Psychologists			3 (4.6)	--
Physiotherapists			1 (1.5)	--
Applied Linguistics			--	29 (100)
Gender				
	Female		49 (75.4)	19 (65.5)
	Male		16 (24.6)	10 (34.5)
Academic education				
	Complete higher education		3 (4.6)	9 (31.0)
	Specialization		24 (36.9)	2 (6.9)
	MSc. in progress		12 (18.5)	3 (10.4)
	MSc.		9 (13.8)	2 (6.9)
	PhD. in progress		5 (7.7)	4 (13.8)
	PhD.		12 (18.5)	9 (31)
Area of activity^†^				
	Outpatient care		32 (49.2)	0 (0.0)
	Primary healthcare		40 (61.5)	0 (0.0)
	Internal medicine		50 (76.9)	0 (0.0)
	Scientific research		13 (20.0)	21 (72.4)
	Teaching consultancy		24 (36.9)	16 (55.1)
	Freelance professional practice^‡^		9 (13.8)	4 (13.7)
Reading frequency in English				
	Once per month		4 (6.1)	0 (0.0)
	Once per week		16 (24.6)	8 (27.5)
	2 or more times per week		24 (36.9)	10 (34.7)
	Every day		21 (32.4)	11 (37.8)
Self-reported knowledge of English				
	Understand			
		A little	2 (3.0)	1 (3.5)
		Reasonably	16 (24.6)	4 (13.8)
		Well	47 (72.4)	24 (82.7)
	Read			
		A little	2 (3.1)	0 (0.0)
		Reasonably	12 (18.5)	2 (6.9)
		Well	51 (78.4)	27 (93.1)
	Speak			
		A little	11 (16.9)	9 (31)
		Reasonably	33 (50.7)	5 (17.3)
		Well	21 (32.4)	15 (51.7)
Total			65 (100.0)	29 (100.0)

*N (%): absolute and relative frequencies

†the indication of more than one area of ​​activity was allowed

‡ the professionals that did not have an employment contract were considered

With regard to the responses to the questions of the questionnaire, the evaluations assigned by the judges and the comments made by them were observed, especially in cases where partial or total reformulation was suggested, in order to identify possible discrepancies between the score given and the opinion of the judge on the question evaluated.

The CVI by category of the instrument and the mean CVI are shown in Table 2.

**Table 2:** Content Validity Index of each section of the protocol, according to the evaluation of the professionals of the Health and Applied Linguistics areas. Belo Horizonte, MG, Brazil, 2015

Sections	Health	Applied Linguistics
1 - Diabetes management plan in the school	0.94	0.90
2 - Blood glucose monitoring	0.94	0.90
3 - Hypoglycemia treatment	0.88	0.90
4 - Hyperglycemia treatment	0.83	0.80
5 - Insulin therapy	0.88	0.90
6 - Food Plan	0.94	0.90
7 - Physical activities and sports	0.88	0.90
Mean CVI (standard deviation)	0.90 (0.042)	0.89 (0.038)

The analysis of the quantification of concordance among the judges of the areas of Health and Applied Linguistics regarding the semantic and idiomatic equivalence gave mean CVIs of 0.9 and 0.89, respectively. It was observed that the mean CVIs of the two groups were very similar, differing by only 0.01. However, the CVI variability in the section of the instrument was higher in the Health area than in the Applied Linguistics area (standard deviation of 0.042 and 0.038, respectively). The category with the lowest acceptance was that of hyperglycemia treatment, for both groups of judges, although with a CVI above 0.78.

The analysis of the comments of the judges by the group of authors of the study allowed the validated version of the instrument to be obtained*. The comments of the judges, considered relevant for the adaptation, were divided into four topics based on the problems raised by the judges, namely: choice of word with broader scope of meaning, inclusion of information relating to the cultural context, choice of lexical items with higher frequency of use in word associations, and explanation of meanings, as described below.

### Choose of word with broader scope of meaning

The term school nurse or trained diabetes personnel had as the translation the synthesis "a trained nurse or professional". In the adapted post-Committee of Judges version, the term chosen was "trained professional", since it is unusual for schools in Brazil to include nurses among their employees. Thus, the term "professional" was chosen because of its wider range of meaning.

### Inclusion of information relating to the cultural context 

Regarding the hypoglycemia treatment, the judges of ​​the Health area suggested the addition of the phone number of the Mobile Emergency Service (SAMU), even though this was present in other sections of the instrument. The inclusion of the guidance to rub sugar or honey in the cheek of the student in cases of severe hypoglycemia was also suggested, since the availability of glucagon is limited in Brazilian schools. Another example concerns the use of an insulin pump, which represents a therapeutic strategy that is little available and restricted to a small number of individuals with diabetes, in Brazil. The same procedure applied to the measurement of ketones in the blood or urine in cases of severe hyperglycemia, since the glucometer apparatus to perform this procedure is limited in its availability.

### Choice of lexical items with higher frequency of use: 

The expressions "counts carbohydrates" and "check blood glucose" of the instrument in English were translated initially as "count the carbohydrates" and "measure the blood glucose". However, the judges of the Applied Linguistics area​​ suggested the translations "measure carbohydrates" and "check the blood glucose", respectively. These suggestions, however, were not incorporated, as "count" and "measure" in combination with "carbohydrate" and "blood sugar", respectively, are associations of words consecrated in the language used by those involved with diabetes care. This would facilitate the understanding by the professionals responsible for the completion of the plan, in the case of the attendant physicians and parents/guardians and for the educators who have access to the instrument in schools. Therefore, the terms "count carbohydrates" and "measure the blood sugar" were ratified in the final version of the instrument, after evaluation by the Committee of Judges.

### Explanation of meanings

The items and expressions that signal, in English, different levels of permission and obligation were translated by equivalent expressions that explain these degrees in Portuguese, in order to avoid possible errors of interpretation. Thus, for example, the statement "may count carbohydrates with supervision" was translated as "the student needs supervision to count carbohydrates", instead of "the student can count carbohydrates with supervision" in order to demonstrate the requirement, in the sense that the students should be supervised, independent of whether he/she has the ability to count carbohydrates.

## Discussion

The Brazilian version of the Diabetes Medical Management Plan, called *Plano de Manejo do Diabetes na Escola*, showed good acceptance among the judges of the areas of Health and Applied Linguistics, with mean CVIs of 0.9 and 0.89, respectively. This facilitated data analysis by the authors of the study, prioritizing the sections that needed changes to the terms and expressions. The strategy of using an interdisciplinary Committee of Judges favored the identification and correction of problems in the translated version and ensured greater semantic, conceptual and technical equivalence of the adapted instrument^12^.

Adequate control of diabetes requires an interdisciplinary approach that goes beyond the limits of the health team, involving environments in which children and adolescents spend a lot of time, such as schools, where it is estimated that children spend approximately one quarter of the hours of the day^14^. However, national and international studies have exposed the unpreparedness of educators and all the school staff to deal with a student with diabetes^15^ ^-^ ^16^ ^).^

Various authors have highlighted the need for documents in schools that contain information regarding the approach of the child and adolescent with diabetes, whose treatment is complex and that requires specific knowledge for the management of the care^7^ ^,^ ^17^ ^-^ ^19^. The *Plano de Manejo do Diabetes na Escola* was developed in order to meet this demand, as it includes individualized guidelines for diabetes treatment to be followed in schools, facilitating the interface between parents, school staff and health professionals^11^_._

This study also presents innovative methodological resources for content adaptation and validation of instruments in the area of ​​Health, namely the interdisciplinary composition of the Committee of Judges, which valorizes the importance of the opinion of each professional, according to his/her area of ​​practice. The consultation through online questionnaire also allowed wider access and more systematization in obtaining and processing the data^20^ ^-^ ^21^.

Some of the advantages highlighted in the literature^22^ ^-^ ^24^ and observed during the application of the questionnaire of the study**_,_** regarding the use of online questionnaire platforms were: (1) convenience, as the respondent can access the questionnaire from anywhere regardless of location, (2) cost reduction, as it is a free tool available on the Internet, (3) multi-disciplinary cover, with broad participation of professionals from different areas, (4) sense of anonymity, opportunity for everyone to express their views individually, (5) speed of obtaining responses, reducing the time of the whole process, and (6) obtaining better quality responses, eliminating transcription errors, that can be exported for analysis into the R statistical program.

One of the limitations of the methodology used in this study concerns the design of the questionnaire and implementation in the electronic platform. In cases where the judges scored the 1- or 2-star options, indicating the need for complete retranslation and partial retranslation with many changes, respectively, the questionnaire was not designed with the space for the comments, in which they could give suggestions for changes or propose a new translation for the excerpt, being obligatory. Thus, the completion was optional and in some cases was not made by the respondent.

The Diabetes Medical Management Plan is available in English and Spanish, and the Portuguese version, the *Plano de Manejo do Diabetes na Escola*, extends its use in the Latin American context^11^. The need to continue the study is highlighted, with testing of the adapted version by the target audience of parents and educators, in order to verify that all sections of the instrument are understandable, and that there is no need to readjust the translated version of the instrument^25^.

## Conclusion

It was concluded that the Brazilian version of the Diabetes Medical Management Plan, named the *Plano de Manejo do Diabetes nas Escolas* (School Diabetes Management Plan), met the criteria of equivalence between the original instrument and the translated version. This provides support for content validation of a Brazilian Portuguese version an instrument of care guidance, to improve the quality of care and knowledge of educators in the treatment of students diagnosed with diabetes, enabling its future use in public and private educational institutions in Brazil.
